# Inhibiting adenoid cystic carcinoma cells growth and metastasis by blocking the expression of ADAM 10 using RNA interference

**DOI:** 10.1186/1479-5876-8-136

**Published:** 2010-12-20

**Authors:** Qin Xu, Xiuming Liu, Wantao Chen, Zhiyuan Zhang

**Affiliations:** 1 Department of Oral and Maxillofacial Surgery, Ninth People's Hospital, Shanghai Jiao Tong University School of Medicine, Shanghai Key Laboratory of Stomatology, Shanghai 200011, China

## Abstract

**Background:**

Adenoid cystic carcinoma is one of the most common types of salivary gland cancers. The poor long-term prognosis for patients with adenoid cystic carcinoma is mainly due to local recurrence and distant metastasis. Disintegrin and metalloprotease 10 (ADAM 10) is a transmembrane protein associated with metastasis in a number of diverse of cancers. The aim of this study was to analyze the relationship between ADAM 10 and the invasive and metastatic potentials as well as the proliferation capability of adenoid cystic carcinoma cells *in vitro *and *in vivo*.

**Methods:**

Immunohistochemistry and Western blot analysis were applied to detect ADAM 10 expression levels in metastatic cancer tissues, corresponding primary adenoid cystic carcinoma tissues, adenoid cystic carcinoma cell lines with high metastatic potential, and adenoid cystic carcinoma cell lines with low metastatic potential. RNA interference was used to knockdown ADAM 10 expression in adenoid cystic carcinoma cell lines with high metastatic potential. Furthermore, the invasive and metastatic potentials as well as the proliferation capability of the treated cells were observed *in vitro *and *in vivo*.

**Results:**

It was observed that ADAM 10 was expressed at a significantly higher level in metastatic cancer tissues and in adenoid cystic carcinoma cell lines with high metastatic potential than in corresponding primary adenoid cystic carcinomas and adenoid cystic carcinoma cell lines with low metastatic potential. Additionally, silencing of ADAM 10 resulted in inhibition of cell growth and invasion *in vitro *as well as inhibition of cancer metastasis in an experimental murine model of lung metastases *in vivo*.

**Conclusions:**

These studies suggested that ADAM 10 plays an important role in regulating proliferation and metastasis of adenoid cystic carcinoma cells. ADAM 10 is potentially an important therapeutic target for the prevention of tumor metastases in adenoid cystic carcinoma.

## Background

Adenoid cystic carcinoma is one of the most common types of salivary gland cancers, characterized by heterogeneous phenotypic features and persistently progressive biological behavior. The poor long-term prognosis for patients with adenoid cystic carcinoma is mainly due to local recurrence related to perineural invasion and delayed onset of distant metastasis, particularly to the lungs[1,2]. In-depth studies on its invasion and metastasis mechanisms are of great significance for the prognosis, evaluation, and selection of treatment protocols.

The ADAM (A disintegrin and metalloprotease) family is a class of type I transmembrane proteins that participate in a wide range of physiological functions. This family of proteins is named because they have two main structural domains, the disintegrin domain and the matrix metalloproteinase domain. They can degrade the extracellular matrix (ECM) and control cell adhesion and movement through regulation of intercellular adhesion, protease activity and cell activities that are closely related to the metastasis of human tumors[3,4]. Among the members of the ADAM family, some ADAMs, such as ADAM 9, 10, 17, are closely involved in the tumorigenesis, development, and metastasis of tumors[5-7]. Recently, ADAM 10 has been reported to play important roles in cell migration, tumor development, and metastasis by proteolytic shedding of cell surface proteins. It has been demonstrated that ADAM 10 can cleave collagen type IV of the basement membrane and is relevant to tumor metastasis[8]. In another study, it was shown that the cleavage of CD44 catalyzed by ADAM 10 contributed to the migration and invasion of glioblastoma tumor cells[9]. In addition, our previous study found that ADAM 10 expression in adenoid cystic carcinoma cells with high metastatic potential was significantly higher than that in adenoid cystic carcinoma cells with low metastatic potential based on gene chip analysis[10]. These findings strongly suggest that ADAM 10 plays an essential role in tumor metastases.

The aim of this study was to analyze the relationship between the expression of ADAM 10 and the invasive and metastatic potentials as well as the proliferation capability of adenoid cystic carcinoma cells *in vitro *and *in vivo*. In the present study, the expression level of ADAM 10 was examined both in primary tumor sections and corresponding metastatic lymph nodes from patients with adenoid cystic carcinoma. RNA interference (RNAi) was applied to inhibit the expression of ADAM 10 in an adenoid cystic carcinoma cell line with high metastatic potential, and the changes in biological behaviors such as cell proliferation and metastasis were observed both *in vitro *and *in vivo*.

## Materials and methods

### Cell lines and specimens

Adenoid cystic carcinoma cells with high metastatic potential (SACC-LM) and low metastatic potential (SACC-83) were provided by the Peking University School of Stomatology[11]. Both cell lines were cultured in RPMI 1640 complete medium with 10% inactivated FBS, 200000 u/L penicillin, and 200000 u/L streptomycin at 37°C. Paraffin specimens of primary foci and metastatic lymph nodes from 15 patients with adenoid cystic carcinoma and cervical lymph node metastasis and paraffin specimens of primary foci of adenoid cystic carcinoma from 20 patients without cervical lymph node metastasis were provided by the Department of Oral Pathology, Ninth People's Hospital, Shanghai Jiao Tong University School of Medicine. The metastatic lymph node tissues were histopathologically graded using a specific three-tier grading system, originally proposed by Szanto et al[12].

### Immunohistochemistry

Immunohistochemistry for ADAM 10 was performed using standard methods. Endogenous peroxidase activity was blocked by treatment with 3% hydrogen peroxide in PBS for 30 min. The specimens were rinsed in PBS. The tissue sections were stained with a mouse monoclonal anti-ADAM 10 antibody (R&D Systems, Minneapolis, MN, USA). The sections were incubated overnight at 4°C (1:50 dilution of primary antibodies). The bound antibody was detected with a secondary biotinylated antibody for 30 min at room temperature and visualized using diaminobenzidine as a chromogenic substrate. The sections were then counterstained with hematoxylin. Immunostaining was defined as positive when more than 30% of tumor cells stained positive. The level of immunostaining was quantified using a semi-automated computerized image analysis system (Image Pro Plus 6.0; Media Cybernetics, Bethesda, FL, USA), which has been successfully applied to analyze histological sections and described in previous reports [13-15]. In brief, the integrated optical density (IOD; IOD = area × average optical density) of positive staining was calculated for each tissue section. The average IOD scores were calculated from triplicate values from each section. The image analysis was performed by three pathologists blinded to the treatment group.

### Preparation of plasmid based ADAM 10 shRNA vector

The ADAM 10 small interfering RNA (siRNA) sequence (CAGUGUGCAUUCAAGUCAA) was designed using the software siRNA Target Designer (Promega, Madison, WI, USA). The preparation of the RNAi vector expressing the human ADAM 10 short hairpin RNA (shRNA) was performed using the pSuper siRNA expression plasmid with the U6 promoter (Oligoengine, Seattle, WA, USA)[16].

### Construction of stable silencing cell lines

SACC-LM cells were transduced with the specific ADAM 10 shRNA vector or an empty plasmid using Lipofectamine 2000 transfection reagent. G418 (300 μg/ml) was used to screen stably transfected clones. The expression of ADAM 10 was examined by real time RT-PCR and Western blotting with an antibody against ADAM 10 (these experiments were repeated three times) to validate the silencing efficiency of the target gene after RNAi. The cell line with stable transfection and effective inhibition of the ADAM 10 gene was named SACC-ADAM 10-RNAi, and the cell line with stable transfection of the control plasmid was named SACC-Mock.

### Quantitative RT-PCR

Quantitative RT-PCR (qRT-PCR) for ADAM 10 transcripts in adenoid carcinoma cell lines was carried out using the PrimeScript RT reagent kit following the manufacturer's instructions (TaKaRa Bio, Shiga, Japa). ADAM 10 gene-specific amplification was confirmed by PCR with specific primers (5'-CTGCCCAGCATCTGACCCTAA-3' and 5'-TTGCCATCAGAACTGGCACAC-3') and subjected to melting curve analysis. GAPDH was used as an internal control for standardization. All qRT-PCR tests were performed in triplicate. The data were analyzed using the comparative Ct method.

### Western blot analysis

Cells were washed twice with cold phosphate-buffered saline (PBS; 137 mM NaCl, 2.7 mM KCl, 10 mM sodium phosphate dibasic, 2 mM potassium phosphate monobasic, pH 7.4) and lysed on ice in buffer (150 mM NaCl, 50 mM Tris-Hcl, 2 mM EDTA, 1% NP-40, pH 7.4) containing protease inhibitors. Equal amounts of protein (20 μg/lane) from the cell lysates were electrophoresed under nonreducing conditions on 10% acrylamide gels. After SDS-PAGE, proteins were transferred to a polyvinylidene difluoride membrane. The membrane was incubated for 2 h in PBS plus 0.1% Tween-20 and 5% nonfat skim milk to block nonspecific binding. Subsequently, the membrane was incubated for 2 h with an antibody against ADAM 10 (R&D Systems, Minneapolis, MN, USA). After washing, proteins were visualized using an ECL detection kit with the appropriate HRP-conjugated secondary antibody (Amersham Pharmacia Biotech, Piscataway, NJ, USA). The membranes were stripped and probed with monoclonal antibodies for GAPDH for loading control as per standard protocols.

### Proliferation assay

The MTT (3-[4,5-dimethylthiazol-2-yl]-2,5-diphenyltetrazolium bromide) colorimetric assay was used to screen for cell proliferation. Briefly, cells were seeded in 8 wells of 96-well plates at a density of 2 × 10^3 ^cells/well. One plate was taken out at the same time every day after the cells had adhered to the wall. Twenty microliters of MTT (5 mg/ml) were added into each well, and the cell culture was continued for 4 h. After aspiration of the medium, the cells were lysed with DMSO. The absorbance was measured using a microplate reader at a wavelength of 490 nm. The measurement was carried out for 8 consecutive days, and the cell growth curve was plotted with OD values as ordinate against time as abscissa. The experiment was repeated three times.

### In vitro invasion assay

Cell invasive behavior was evaluated using 24-well transwell units with 8-μm porosity polycarbonate filters. The filters were coated with 50 μl of 8 mg/ml reconstituted basement membrane substance (Matrigel; BD Biosciences, San Diego, CA, USA). The coated filters were air-dried at 4°C prior to the addition of the cells. The basement membrane was hydrated with 50 μl serum-free RPMI 1640 medium 30 min before use. The cells were digested with trypsin, and the cell density was adjusted to 1 × 10^6^/ml using serum-free RPMI 1640 medium. A total of 200 μl of cell suspension was added into each upper Transwell chamber, and 600 μl of RPMI 1640 medium containing 5% fetal bovine serum was added into the lower chamber. There were three duplicates for each cell group. Then, the cells were incubated for 24 h in a humidified atmosphere of 5% CO_2 _at 37°C. Cells were fixed with methanol and stained with Giemsa. Cells on the upper surface of the filter were removed by wiping with a cotton swab, and invasion was determined by counting the cells that migrated to the lower side of the filter with optical microscopy at 400×. A total of five visual fields at the center and in the surrounding areas were counted, and the average was calculated[17]. The experiment was repeated three times.

### Analysis of lung metastasis in vivo

Four-week-old female BALB/c nu/nu nude mice were raised under specific pathogen free conditions. All animal experiments were carried out according to the standards of animal care as outlined in the Guide for the Care and Use of Experimental Animals of the Medical College of Shanghai Jiaotong University. The study protocol was approved by the hospital ethical committee.

As an experimental lung metastasis model, 0.2 ml single-cell suspensions (10^6 ^cells) were injected via the mouse tail vein. There were seven mice in each group. The mice were sacrificed 40 days after inoculation, and bilateral lung tissues were removed. Pathological sections of lung tissues with the maximum cross-sectional area were prepared. Tumor burden was determined by weighing the lungs of the animals as described in previous reports[18-20].

### Statistical analysis

A Fisher's exact test was performed to compare differences in ADAM 10 expression levels between primary tumors and corresponding metastatic lymph node groups. Normally distributed, continuous variables were compared using one-way analysis of variance (ANOVA). When ANOVA produced a significant difference between groups, multiple comparisons of group means were performed using the Bonferroni procedure with a type I error adjustment. Repeated measure analyses were performed to assess the group effects on proliferative capacity over the time course. Data are presented as mean ± standard deviation. All statistical assessments were two-sided and evaluated at the 0.05 significance level. All statistical analyses were performed using SPSS 13.0 statistics software (SPSS, Chicago, IL, USA).

## Results

### ADAM 10 expression in primary and metastasized adenoid cystic carcinoma tissue samples

**Figure 1:**
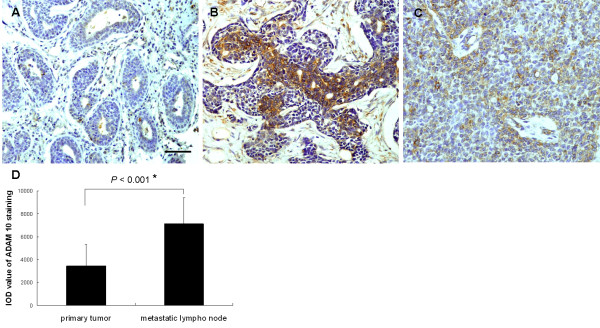
**Immunohistochemical staining for ADAM 10 on paired primary adenoid cystic carcinoma (a) and corresponding metastatic lymph nodes (b) and in 20 primary foci tissues without cervical lymph node metastasis (c)**. Scale bar = 100 μm. (d) The IOD value of ADAM 10 staining (mean ± SD) in metastatic lymph nodes was significantly higher than that in primary tumors (*p < 0.001).

First, ADAM 10 expression was examined by immunostaining of 15 paired tissues from patients with oral adenoid cystic carcinoma and cervical lymph node metastasis. For each pair of tissues, primary tumor sections and corresponding metastatic lymph nodes were examined. ADAM 10 was only detected in 26.7% of primary tumors (4/15; Figure 1A), whereas 80% of corresponding metastatic lymph nodes showed positive ADAM 10 staining (12/15; Figure 1B). Table 1 shows the overall ADAM 10 expression in metastatic lymph nodes according to the histologic grade, which indicated that the ADAM 10 immunoreaction was stronger with a higher histologic grade. The Fisher's exact test indicated that the expression levels of ADAM 10 in corresponding metastatic lymph nodes were statistically higher than those in the primary tumors (p = 0.004). The IOD value of ADAM 10 staining for metastatic lymph nodes was also significantly higher than the ADAM 10 staining for primary tumors (p < 0.001; Figure 1D), suggesting that ADAM 10 expression is closely related to tumor metastasis. Next, ADAM 10 expression in 20 primary foci tissues without cervical lymph node metastasis were detected. In these cases, 30% of primary tumors (6/20) showed positive staining (Figure 1C), which indicated a similar expression rate in primary foci.

**Table 1:** ADAM 10 expression in metastatic lymph nodes according to the histologic grade

	ADAM 10 expression				
**Grade**	**Negative No. (%)**		**Positive No. (%)**		**Total**

I	0	0	0	0	0

II	1	33.3%	3	25%	26.7%

III	2	66.7%	9	75%	73.3%

### ADAM 10 expression in adenoid cystic carcinoma cells with different metastatic potentials

**Figure 2:**
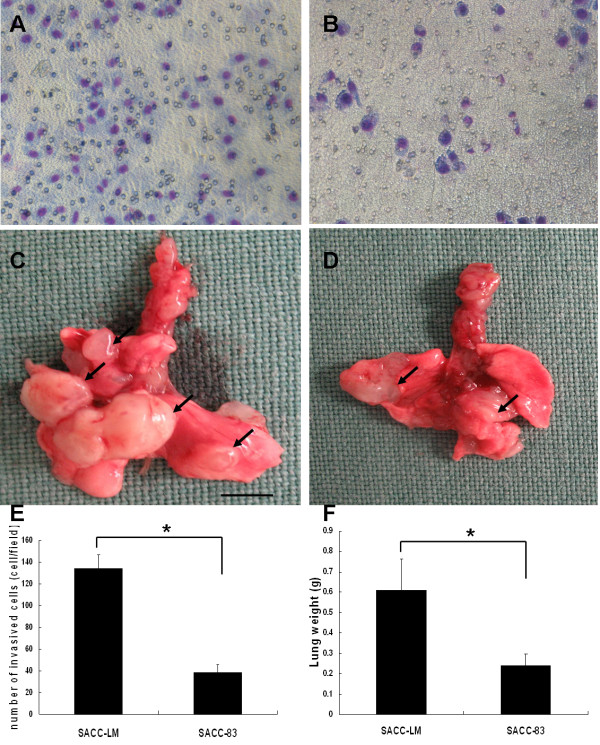
**Detection of the metastatic potential of SACC-LM and SACC-83 cells**. (a), (b) A Matrigel transwell invasion assay was used to test the ability of SACC-LM and SACC-83 cells to invade the filter membrane. (c), (d) Overview of lung tissues from mice injected with SACC-LM and SACC-83 cells (scale bar = 0.5 cm). Tumors are indicated by black arrows. (e) Values represent the cell number (mean ± SD) per visible field (*p < 0.001). (f) Lung weight (*p < 0.001).

The metastatic potential of SACC-LM and SACC-83 cells was investigated using a matrigel invasion assay and experimental lung metastasis tests. The invasion assay results indicated that SACC-LM cells had a significantly higher ability to pass through the basement membrane compared to SACC-83 cells (p < 0.001; Figure 2A, B, E). Similarly, the experimental lung metastasis results (n = 7 mice per group) showed the lung weight derived from SACC-LM group was 0.61 ± 0.15 g, compared to 0.24 ± 0.06 g from the SACC-83 group (p < 0.001; Figure 2C, D, F). These results verified the difference in metastasis potential of SACC-LM and SACC-83 both*in vitro *and*in vivo*.

**Figure 3:**
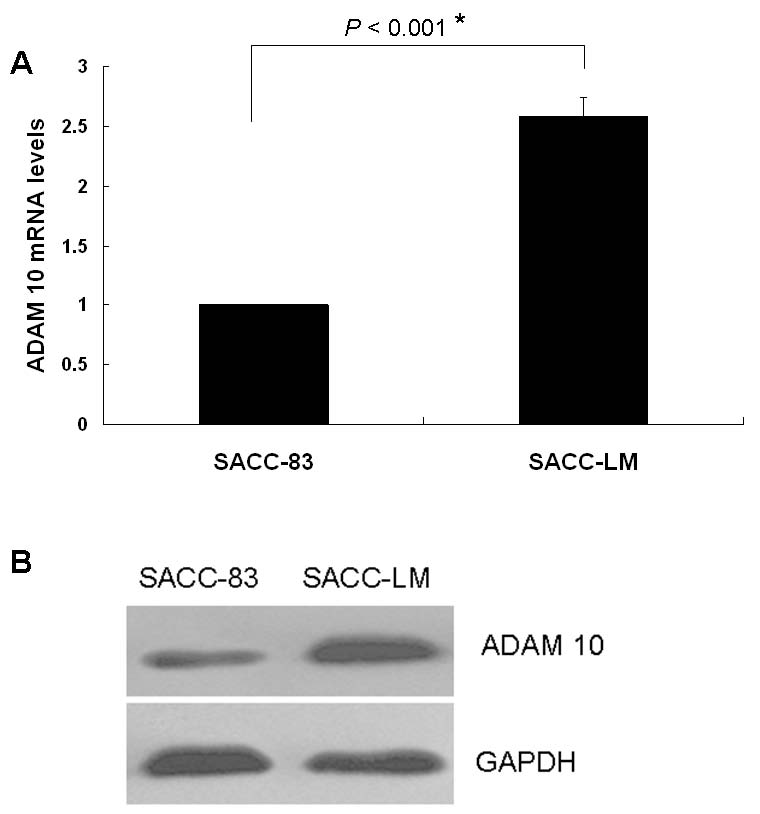
**ADAM 10 expression levels in SACC-83 and SACC-LM cell lines**. (a) Quantitative RT-PCR showing relative ADAM 10 mRNA levels (mean ± SD) in SACC-83 cells (low metastatic potential) compared with SACC-LM cells (high metastatic potential) (*p < 0.001). (b) Western blot analysis showing ADAM 10 protein expression in SACC-83 and SACC-LM cell lines. GAPDH served as a loading control.

Subsequently, both ADAM 10 mRNA and protein levels were examined in adenoid cystic carcinoma cells with either high (SACC-LM) or low (SACC-83) metastatic potential. ADAM 10 was more abundant at both the mRNA and protein level (about 2.6 fold) in SACC-LM cells when compared to SACC-83 (Figure 3A and 3B), which corroborated the tumor tissue results and indicated that ADAM 10 overexpression might correlate with cancer metastasis.

### Abolished ADAM 10 expression in SACC-LM cells

**Figure 4:**
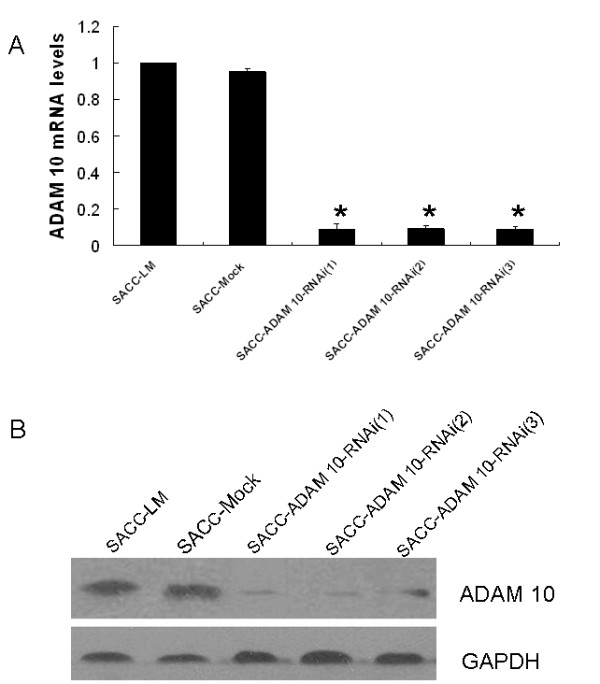
**Abolishment of ADAM 10 expression in SACC-LM cells**. (a) ADAM 10 mRNA levels were determined by qRT-PCR. Relative fold induction for the ADAM 10 mRNA (mean ± SD) in mock- and ADAM 10 siRNA-transfected cells is presented relative to the expression in parental SACC-LM cells (*p < 0.001 compared with SACC-LM). (b) Western blot analysis for ADAM 10 protein expression in the indicated cell lines. GAPDH was used as a loading control. SACC-LM (high metastatic potential control); SACC-Mock (mock transfection control); SACC-ADAM10-RNAi (1), (2), and (3) represent the three different clones, respectively.

To investigate whether ADAM 10 expression was essential for the metastatic capability of SACC-LM cells, stable ADAM 10 RNAi transfected cells (SACC-ADAM10-RNAi) and a mock-transfected control cell line (SACC-Mock) were established as described above. Three cellular clones with stable ADAM 10 RNAi transfection, SACC-ADAM10-RNAi (1), (2), and (3), were selected for further evaluation. Compared to parental (SACC-LM) and mock-transfected (SACC-Mock) cells, both mRNA and protein expression of ADAM 10 were significantly reduced in SACC-ADAM10-RNAi (1), (2), and (3) cells (all, p < 0.001; Figure 4A, B).

### Gene silencing of ADAM 10 reduces cell proliferation and migration in SACC-LM cells

**Figure 5:**
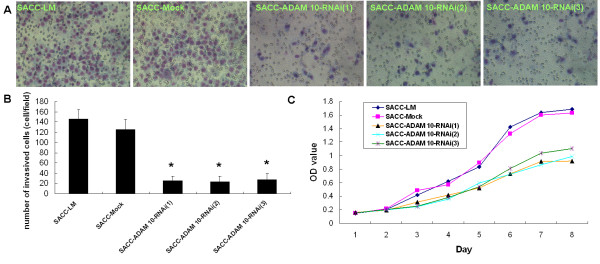
**Gene silencing of ADAM 10 reduces cell proliferation and migration in SACC-LM cells**. (a) A Matrigel transwell invasion assay was used to test the ability of the indicated cell lines to invade the filter membrane. (b) Values represent the cell number (mean ± SD) per visible field (*p < 0.001 compared with SACC-LM). (c) Cell proliferation was analyzed using the MTT assay. Cells were monitored for 8 days and the average OD490 (± SD) for each cell line is shown. Cells transfected with ADAM 10 siRNA showed reduced cell growth relative to parental and mock-transfected cells. SACC-LM (high metastatic potential control); SACC-Mock (mock transfection control); SACC-ADAM10-RNAi (1), (2), and (3) represent the three different clones, respectively.

To examine whether the knockdown ADAM 10 expression had any effect on cell growth, an MTT cell proliferation assay was performed. Compared to parental (SACC-LM) and mock-transfected (SACC-Mock) cells, ADAM 10-RNAi cells showed decreased cell proliferation, supporting the role of ADAM 10 in cell growth in SACC-LM cells (Figure 5C). In addition, the affect of gene silencing of ADAM 10 on the cell migration ability of SACC-LM cells was also investigated by transwell invasion assay (Figure 5A). The results indicated that ADAM 10-RNAi cells had a significantly reduced ability to pass through the basement membrane when compared to the parental and mock-transfected cells (all, p < 0.001; Figure 5B). These data supported the notion that ADAM 10 expression is essential for both cell proliferation and migration.

### Gene silencing of ADAM 10 reduces tumor metastasis in vivo

**Figure 6:**
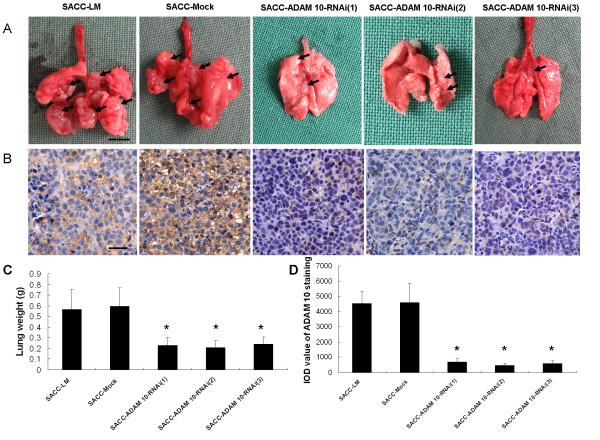
**Gene silencing of ADAM 10 reduces tumor metastasis in vivo**. (a) Overview of lung tissues from mice injected with the indicated cell lines (scale bar = 0.5 cm). Tumors are indicated by black arrows. (b) Immunohistochemical staining of ADAM 10 from tumors derived from injected SACC-LM cells (scale bar = 50 μm). (c) Lung weight. (d) Quantification of immunohistochemical staining of ADAM 10 from b using Image Pro Plus software (*p < 0.001 compared with SACC-LM). SACC-LM (high metastatic potential control); SACC-mock (mock transfection control); SACC-scrambled RNA (scrambled siRNA control); SACC-ADAM 10-RNAi (1), (2), and (3) represent the three different clones, respectively.

To evaluate if ADAM 10 expression was essential for the metastatic potential of SACC-LM cells *in vivo*, parental (SACC-LM), mock-transfected SACC-LM cells (SACC-Mock), or ADAM 10-RNAi SACC-LM cells-SACC-ADAM 10-RNAi (1), (2), and (3)-were injected into BALB/c nude mice (n = 7 mice per group). Mice were sacrificed 40 days after inoculation, and their bilateral lung tissues were removed and subjected to histological examination (Figure 6A). The lung weights derived from parental and mock-transfected SACC-LM cells were 0.57 ± 0.19 g and 0.60 ± 0.17 g, respectively, compared to 0.23 ± 0.08 g, 0.21 ± 0.07 g, and 0.24 ± 0.07 g for the SACC-ADAM 10-RNAi (1), (2), and (3) groups. The lung weight test revealed a significant reduction of tumor burden in ADAM 10-RNAi cells as compared to parental or mock-transfected SACC-LM cells (p < 0.001; Figure 6C). Next, ADAM 10 expression in these tumors was examined. As expected, ADAM 10 expression was severely reduced in tumors derived from ADAM 10-RNAi cells compared to tumors derived from parental or mock-transfected cells (Figure 6B, D). These data again supported the argument that ADAM 10 is essential for metastasis in adenoid cystic carcinoma.

## Discussion

A variety of ADAMs including ADAM 10 have been shown to be overexpressed in cancers, and it has been hypothesized that the downregulation of ADAM 10 may suppress tumor growth and metastasis in adenoid cystic carcinoma. However, previous reports that may relate to this hypothesis are very limited. The purpose of this study was to analyze the relationship between the gene silencing of ADAM 10 and the invasive and metastatic potentials as well as the proliferation capability of adenoid cystic carcinoma cells *in vitro *and *in vivo*.

In this study, we have characterized the expression of ADAM 10 in adenoid cystic carcinoma tissues. Immunohistochemical analysis indicated that ADAM 10 expression was significantly elevated in metastatic lymph nodes compared with corresponding primary tumors, and ADAM 10 immunoreactivity was stronger with a higher histologic grade in metastatic lymph nodes. In addition, both mRNA and protein levels of ADAM 10 were more abundant in an adenoid cystic carcinoma cell line with high metastatic potential (SACC-LM) than in a cell line with low metastatic potential (SACC-83). This result indicated that high ADAM 10 expression tends to occur in metastatic tumor tissues and overexpression of ADAM 10 might be a potential prognostic sign of high metastatic risk, which is consistent with prior studies. Lee et al. reported that ADAM 10 was upregulated in melanoma metastases compared with primary melanomas[21]. In another study, Gavert et al. reported that the expression of ADAM 10 was detected at the invasive front of human colorectal tumor tissues[22]. Based on these data, it is reasonable to speculate that ADAM 10 may play a role in tumor invasion and metastasis.

To provide evidence supporting this supposition, we investigated the effects of ADAM 10 silencing on *in vitro *cell invasion as well as *in vivo *cancer metastasis in an experimental murine model of lung metastasis. The expression of ADAM 10 was specifically knocked down in human adenoid cystic carcinoma cell lines with high metastatic potential using RNAi. Downregulation of ADAM 10 resulted in a suppression of tumor cell invasion *in vitro *and decreased experimental lung metastasis *in vivo*, which strongly supported that ADAM 10 is involved in the process of tumor metastasis. Our finding is in agreement with previous reports on the functional roles of ADAM 10. As we know, to metastasize, malignant cells must first detach from the dense, cross-linked collagen network of the ECM and migrate through the host vasculature before extravasating the vasculature and infiltrating the host tissues [23]. Therefore, tumor metastasis is dependent on the tumor's ability to degrade the surrounding ECM and reduced cell adhesion. A number of studies have demonstrated that the metalloprotease domain of ADAM 10 can cleave and remodel ECM proteins such as type-IV collagen and CD44[24] and influence cell-cell signaling, including the Notch pathway[25,26]. The disintegrin domain of ADAM 10 can also interact with matrix adhesion molecules. Hence, ADAM 10 is able to modulate a variety of cell-cell and cell-ECM interactions and consequently digest the basement membrane, facilitate cell migration, and promote tumor metastasis. However, the detailed mechanism by which ADAM 10 interacts with ECM proteins is not very clear. Further studies are required to determine these exact mechanisms. Moreover, in our study, downregulation of ADAM 10 expression significantly inhibited experimental lung metastasis, which suggested this therapy might be a novel and promising treatment strategy for metastasis.

In addition, in the present study, the transfection of ADAM 10 siRNA resulted in a significant reduction of cellular growth of adenoid cystic carcinoma cells. Our data are in line with previous reports showing that ADAM 10 expression is correlated with the proliferation of tumor cells. Lee et al. demonstrated that the expression of ADAM 10 correlated with increased melanoma cell proliferation[18]. Similarly, Ko et al. confirmed the effects of ADAM 10 on the growth of oral squamous cell carcinoma cells[27]. In another study, results indicated that suppression of ADAM 10 expression leads to a significant decrease in prostate cell growth[28].

This effect on growth promotion might also be related to its protease activity. It has been demonstrated that ADAM 10 can cleave amyloid precursor protein[29-31], a critical transmembrane molecule related to the growth of several types of cells[32-34], which suggests that ADAM 10 may influence the proliferation of adenoid cystic carcinoma cells via amyloid precursor protein shedding. Furthermore, Ko et al. reported that ADAM 10 could inhibit oral squamous cell carcinoma cell growth through its α-secretase activity[27]. Jin et al. have indicated that ADAM 10 can active Notch signaling by suppressing ectodomain shedding of delta-1, which subsequently leads to a strong inhibitory effect on tumor cell proliferation[35]. These studies reveal that different mechanisms seem to be involved in the anti-proliferative effects of ADAM 10 against tumor cells. Importantly, in the present study, we discovered a significant growth inhibition of adenoid cystic carcinoma cells following downregulation of ADAM10 via ADAM 10-specific siRNA, which suggested that ADAM 10 is a promising new therapeutic target for the treatment of adenoid cystic carcinoma.

## Conclusions

Collectively, our data suggested that ADAM 10 expression is closely associated with adenoid cystic carcinoma metastasis. Reduced ADAM 10 expression not only impacted cell proliferation, but it also decreased the metastatic potential of adenoid cystic carcinoma cells. Thus, ADAM 10 is a potential therapeutic target for the treatment of adenoid cystic carcinoma.

## Competing interests

The authors declare that they have no competing interests.

## Authors' contributions

QX participated in the design of the study, carried out the immunohistochemistry, Western blot analysis, performed the statistical analysis, and drafted the manuscript. XL participated in animal sacrifice. WC carried out proliferation and invasive analyses. ZZ conceived the study and participated in its design. All authors have read and approved the final manuscript.
